# rTLS: Secure and Efficient TLS Session Resumption for the Internet of Things †

**DOI:** 10.3390/s21196524

**Published:** 2021-09-29

**Authors:** Koen Tange, Sebastian Mödersheim, Apostolos Lalos, Xenofon Fafoutis, Nicola Dragoni

**Affiliations:** DTU Compute, Department of Applied Mathematics and Computer Science, Technical University of Denmark, Richard Petersens Plads, 2800 Kongens Lyngby, Denmark; samo@dtu.dk (S.M.); lalosapost@gmail.com (A.L.); xefa@dtu.dk (X.F.); ndra@dtu.dk (N.D.)

**Keywords:** network, security, protocol, formal verification

## Abstract

In recent years, the Transport Layer Security (TLS) protocol has enjoyed rapid growth as a security protocol for the Internet of Things (IoT). In its newest iteration, TLS 1.3, the Internet Engineering Task Force (IETF) has standardized a zero round-trip time (0-RTT) session resumption sub-protocol, allowing clients to already transmit application data in their first message to the server, provided they have shared session resumption details in a previous handshake. Since it is common for IoT devices to transmit periodic messages to a server, this 0-RTT protocol can help in reducing bandwidth overhead. Unfortunately, the sub-protocol has been designed for the Web and is susceptible to replay attacks. In our previous work, we adapted the 0-RTT protocol to strengthen it against replay attacks, while also reducing bandwidth overhead, thus making it more suitable for IoT applications. However, we did not include a formal security analysis of the protocol. In this work, we address this and provide a formal security analysis using OFMC. Further, we have included more accurate estimates on its performance, as well as making minor adjustments to the protocol itself to reduce implementation ambiguity and improve resilience.

## 1. Introduction

There are many examples of well-established communication protocols that are able to satisfy contextually-defined requirements and are in use in modern technology. Arguably the most well-known example is the TLS protocol [1]. This protocol is widely used in today’s Internet, although originally designed for the Web. Recently, this protocol has been gaining traction in the IoT domain, as well. To better suit the heterogeneous needs present in this domain, adaptions and new extensions of the TLS protocol are needed, specifically to enable extremely lightweight devices to partake in TLS connections, as well.

While securely browsing the Web, it is not unusual for a TLS handshake to require between 1 and 4 KB of traffic. For consumer devices with browsers, this is often not an issue, but it is a lot of traffic overhead for lightweight devices running on battery power, where powering a wireless radio is very costly. Therefore, there is a need to reduce this handshake overhead as much as possible. To reduce bandwidth overhead, as well as latency, TLS 1.3 features a new zero round-trip time (0-RTT) session resumption protocol capable of transmitting application data already in its first flight of messages. This allows for the quick reopening of a session without having to go through an expensive full handshake again. Unfortunately, this resumption protocol is susceptible to replay attacks, a design decision deemed acceptable by the TLS committee since web connections usually start an HTTP GET request, which is idempotent. For IoT applications, however, this assumption does not hold. For example, consider a temperature sensor that periodically reports its readings to a server. This is our primary motivation for extending the 0-RTT protocol with an IoT-friendly alternative.

As a secondary goal, we aim to further reduce traffic overhead of the resumption handshake. The reason for this is that the financial costs of sending TLS handshakes for every periodic IoT transmission could increase rapidly. For example, it is expected that many network providers for 5G and Low-Power Wide Area Networks (LPWAN) will charge their users based on data usage [2,3,4]. Additionally, the transmission cost of setting up a secure connection should be within reasonable proportions to the size of the payload itself. If it is tens of times higher, users might choose not to use secure channels, or alternatively implement their own cryptographic protocols, with associated risks.

The standard TLS protocol is designed with the assumption in mind that servers do not keep state on a client in between sessions. This is justifiable for the Web, where the set of potential clients is unbounded and it is often hard to predict if a client will resume a session at all. However, for many IoT systems, it is reasonable to assume that the set of clients is fairly static and known a priori, or otherwise traceable through a key infrastructure. Thus, keeping state on these clients in between sessions is a lot easier than for the Web, and having state information at the ready can aid in further reducing handshake overhead. TLS 1.3 does not offer any such mechanism, leaving this as a gap that can be filled by IoT-friendly extensions.

In previous work, we introduced rTLS [5], a TLS extension that can authenticate two endpoints and set up a secure connection with minimal additional overhead, given that the client and server have initiated a session in the past. We described how the extension changes the 0-RTT session resumption protocol to reduce overhead compared to the standard protocol, while adding new security features including replay protection, forward secrecy, and break-in protection. We built the protocol on the assumption that servers can store state on clients, with the IoT in mind. Additionally, we provided equations on the lower bound for traffic overhead of any TLS resumption protocol, as well as our proposed extension, and compared it to overhead observed from the OpenSSL [6] implementation of TLS 1.3. We also provided estimations for storage overhead for both client and server.

In this work, we extend upon the original work on multiple points. The first and main extension point is the addition of a formal analysis of protocol security using the Open-source Fixed-point Model Checker (OFMC) [7], including the development of a new intermediate specification language to help with this verification. To this end, the original text covering security analysis has been completely revised and is included in a new separate section, Section 4. Other points include the addition of more accurate performance and storage overhead estimates, based on better observations and a better understanding of TLS implementations. The protocol itself has also received some minor updates relating mainly to which data is stored between sessions. Further, we have made minor improvements to the presentation of the protocol design section.

The remainder of this paper is organized as follows: In Section 2, we briefly discuss the foundations necessary to understand our proposed extension. We then explain our extension in detail in Section 3. After that, we provide a formal analysis of several security properties in Section 4. Then, we evaluate the storage and transmission overhead in Section 5, after which we discuss related work in Section 6. Finally, we conclude in Section 7.

## 2. Preliminaries

In this section, we briefly discuss the essentials needed to understand the concepts upon which our proposed solution is built. First, we summarize the TLS 1.3 protocol, after which we discuss the Double Ratchet algorithm and Key Derivation Function (KDF)-chains. Both of these are described in more detail in the the Signal documentation pages [8].

### 2.1. TLS 1.3

The TLS 1.3 protocol [1] establishes a secure communication channel (a session) between a client (the initiator) and a server. The most common use establishes one-way authentication; only the server is authenticated, using a key distribution method, such as certificate authorities.

In the most typical scenario, one-way authentication is provided, that is, the server authenticates itself to the client, building on the certificate authority paradigm for key distribution. The protocol also supports session resumption, allowing users to quickly renegotiate a session in fewer round-trips, leveraging state data from past sessions between those two users. In this section, we only briefly discuss necessary elements of the protocol. For a more in-depth discussion, we refer to the standard [1].

In order to speed up session negotiation, TLS 1.3 provides several improvements over its predecessor, TLS 1.2 [9]. One of the major improvement points is the introduction of 0 Round-Trip Time session resumption, or 0-RTT. The defining feature of 0-RTT resumption is that application data can already be transmitted to the server in the first message sent by the client. The standard refers to this as early data. The standard specification comes with a caveat: early data must be idempotent, that is, it should result in a state change on the server. This is because 0-RTT handshakes are susceptible to replay attacks.

The 0-RTT protocol is set up as follows: After the initial (non-resumption) handshake, 0-RTT key data is transmitted to the client in a NewSessionTicket message. The message contains a ticket, as well as other data later needed by the server to continue a session. When the client later initiates a session resumption, it will send this ticket to the server as part of the first message, enabling the server to continue the session without needing a full TLS handshake. Note that, while the standard describes a structure for NewSessionTicket messages, it does not not prescribe a specific structure for the tickets which these encapsulate, essentially leaving room for a variety of implementations from, e.g., databases with lookup keys to self-encrypted and authenticated messages. In this work, we assume the mechanism first introduced in RFC 5077 [10], a solution optimized for the Web, and which requires no server-side state variables on closed sessions. This method has already seen use in TLS 1.2 and is supported by many TLS libraries, such as OpenSSL [6] and WolfSSL [11]. With this approach, the ticket contains all state variables needed by the server and is encrypted with a key known only to the server. From the client’s perspective, it receives an opaque blob of encrypted data. When the client initiates session resumption, it will send over this ticket, which can then be decrypted by the server, enabling it to restore the session state.

### 2.2. Double Ratchet Algorithm

The Double Ratchet Algorithm [8] is a cryptographic protocol enabling highly secure, asymmetric message exchange between multiple parties. The protocol was originally developed for Signal [12] but is now also used in the popular messaging app WhatsApp [13]. It has received significant cryptographic attention and has been formally verified [14].

At the heart of this protocol lies a KDF-chain. This is a KDF function that can be iteratively applied to its own output, creating a feedback loop where part of the output of each iteration is fed back into the function as input for the next iteration, while also providing key material for encrypting messages. This construction is commonly referred to as a ratchet because of the one-way nature of the KDF function; new keys can be generated constantly, but one cannot reverse the process to produce old keys. Because of this, when used correctly ratchets are resilient against replay attacks and can provide forward secrecy.

**Figure 1:**
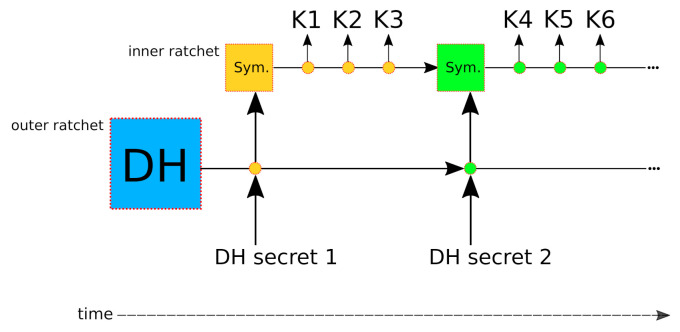
The double ratchet process and structure. Rectangles indicate initial states, circles indicate “spins” of the ratchets, and colors indicate the flow of entropy from a DH exchange. The outer ratchet is depicted on the bottom, with the inner ratchet above it.

A double ratchet is a combination of multiple ratchet-like constructions. Firstly, it contains one “outer” ratchet, and secondly one or more “inner” ratchets. These ratchets work together to provide stronger security properties. The outer ratchet receives periodic (e.g., every 10 messages) external entropy from a Diffie-Hellman (DH) handshake as part of its input. Whenever this outer ratchet is spun (i.e., its KDF function is invoked), its output includes new input keys for the inner ratchets. These inner ratchets are reset completely and seeded with the new input keys. This provides post-compromise, or break-in, protection. One can also spin just one of the inner ratchets to produce keys that can be used to encrypt or decrypt messages. The outer ratchet is also commonly referred to as the DH ratchet, and the inner ratchets are often called symmetric ratchets (because their input keys are symmetric). Figure 1 illustrates the ratchet process. As can be seen in the figure, with the progression of time, multiple symmetric ratchets may be instantiated in succession (or, in other words, the same inner ratchet is reset whenever the outer ratchet is spun). The first inner ratchet, producing keys K1,K2, and K3 received entropy from the first DH handshake (this is visualized by a yellow color). When the outer ratchet is spun a second time, the inner ratchet will be reset and receive fresh entropy from the outer ratchet, which we emphasize with a green color, to indicate that the inner ratchets have different entropy.

In the standard Double Ratchet Algorithm, both parties maintain one DH ratchet and two symmetric ratchets, respectively, for outgoing incoming messages. In our work, we use only one symmetric ratchet, as only the client will ever initiate a connection, and, thus, the client will only need a ratchet for sending, while the server only needs one for receiving. For more details on the double ratchet algorithm, we refer the reader to Reference [8].

## 3. Ratchet TLS (rTLS)

In this section, we specify our proposed extension, ratchet TLS (rTLS). Note that we have designed the specification with the following design goals in mind: firstly, to maximize the use of existing extensions and utilities in the TLS suite; secondly, to require only minimal changes to those parts that are changed; thirdly to minimize bandwidth overhead; and, finally, to provide stronger 0-RTT security properties.

Our extension relies on a Symmetric Ratchet to generate the encryption keys for early data encryption in session resumption. Further, it builds on standard TLS extensions to provide an (outer) DH ratchet, providing forward secrecy and break-in protection. We can elegantly transmit the data relevant to our new extension as a Pre-Shared Key (PSK), and can signal support for rTLS by making use of the psk_key_exchange_modes extension specified in RFC 8446 [1]. As the following sub-sections will show, the changes necessary to the TLS protocol to achieve this are kept to a minimum.

We will first discuss the differences between standard TLS handshakes and ratchet-mode handshakes. First, we will discuss the changes to the initial handshake in Section 3.1, after which we look at the differences for the resumption handshake in Section 3.2. Finally, we specify the protocol setup and operation in detail in Section 3.3.

### 3.1. Initial Handshake

**Figure 2:**
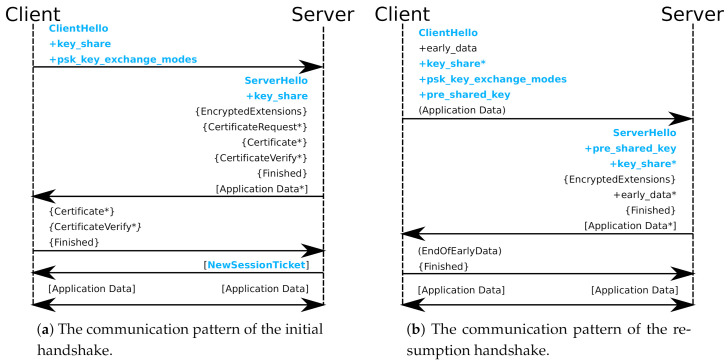
(**a**,**b**) The initial resumption handshake communication patterns, respectively. + denotes an extension, and * denotes an optional or situational component, while {} and [] denote encryption with a derivation of the handshake or application secret, respectively. Modifications from the original handshakes are printed in blue.

Figure 2a shows the communication pattern of the initial handshake of a typical rTLS-enabled TLS sessions. For ease of comparison with the RFC [1], we utilize the same syntax and have adopted the common extensions depicted in the standard. The communication pattern of this handshake looks identical to a standard TLS handshake. This is because no new extensions are added. Rather, we further extend the existing PSK-related extensions. In the figure, the extensions that are affected by rTLS are denoted in blue.

In the first flight of messages, the psk_key_exchange_modes extension is included by the client to tell the server that it wants to obtain a session ticket. The client and server must agree on a KDF and which ciphers to use for resumption. In principle, any secure KDF and cipher can be used; however, to keep the number of protocol changes to a minimum, we reuse the ciphers included in the cipher-suite, agreed upon by both parties through the TLS handshake. This way, we can ensure that both client and server support the chosen ciphers. Additionally, this makes reasoning about the protocol easier because we only have to consider one type of KDF. Note that the agreement on which cipher-suite to use is only finalized after the server has sent its ServerHello message. The secret key derived from the DH handshake conducted through the key_share extension is used in the derivation of all secrets used in TLS, including the PSK resumption secret. Thus, whenever a key_share extension is part of a handshake, a fresh entropy source is introduced into the key schedule. The psk_key_exchange_modes extension consists of a list of a byte-sized enumerated type. This type indicates the PSK mode to use. Currently, TLS supports value 0 for a static PSK and 1 for an (EC)DHE established PSK. We extend this by adding another value indicating a PSK with key ratcheting. This list of PSK modes advertises which types the client supports to the server.

After finalizing the initial handshake, the server sends a NewSessionTicket to the client. The specification explicitly allows for sending multiple tickets in one session, although this is not necessary, since session resumption by itself can add fresh entropy when needed (through a DH handshake), thereby introducing freshness into resumption tickets at a later time. All fields specified in the TLS specification for the NewSessionTicket structure are listed in Table 1.

Since the specification enables the ticket field to carry opaque binary data, we specify it to include a 4-byte “connection identifier” that the server can later use to uniquely identify the session so that it may access the locally stored state for that session. Note that it does *not* include a shared resumption key. The standard defines the resumption key as being derived from the ticket nonce and TLS master secret. Further, we include a fresh DH public key generated by the server, which the client will use to initialize its DH ratchet for the first resumption. In later resumptions, DH parameters can be shared through the key_share extensions; however, for the very first resumption, we have to make an exception since the client needs to be able to initialize the ratchet. The extensions field should include the early_data extension, which tells the client that this PSK can be used to transmit early data.

### 3.2. Session Resumption

Figure 2b shows the resumption handshake communication pattern. Again, it looks indistinguishable from a standard TLS 0-RTT resumption handshake, but the elements noted in blue text indicate that they deviate in usage or content in rTLS.

Firstly, the client can optionally include a key_share extension. This is not necessary for every resumption handshake, and the exact frequency with which these should be included depends on the desired granularity of break-in resilience; if it is included in every handshake, then break-in recovery occurs with every resumption, while including it only every *n* resumption handshakes will imply break-in recovery every *n* resumptions and so on. We refer to the frequency with which *n* is included as the DH exchange period. If the server receives a key_share from the client, it will reply with a key_share of its own, to complete the DH handshake.

Secondly, the client includes a psk_key_exchange_modes extension indicating which PSK mode is used for the pre_shared_key field. This should be set to the enumerated type value representing rTLS.

The pre_shared_key field contains the connection identifier, which the server can identify this session, as well as the current ratchet index used by the client. Based on this index, the server can determine if it missed any previous resumption attempts and spin its ratchet enough times to catch up and ensure the encryption keys are synchronized with the client. The pre_shared_key contains a list of PskIdentity structures, as well as a list of PskBinderEntry structures. Each entry in the PskIdentity consists of an identity value and a obfuscated_ticket_age value. We do not make any changes to the ticket age, and refer to the standard for details on how to derive the obfuscated ticket age. The identify field is defined as opaque binary data, which allows us to use it to transmit the 4-byte connection ID that was transmitted by the server in the initial handshake, as well as a 1-byte ratchet index representing the current index (after having derived the latest resumption master secret) of the symmetric KDF-chain. The PskBinderEntry list is a list of Hash-Based Message Authentication Code (HMAC) values which authenticate the handshake from the ClientHello up to (and including) the list of PSKIdentity entries.

The client spins its symmetric ratchet whenever it initiates a resumption handshake, thereby ensuring that the resumption master secret changes all the time. As described in the standard, an early traffic is derived from the resumption master secret, which, in turn, is used as an encryption key for the early data. The server can decrypt this early data once it has received a ClientHello with the necessary extensions for ratchet-mode resumption. It is then able to access the ratchet state for the given connection ID and spin this ratchet until it is equal to the received ratchet index, thereby obtaining the keys necessary to decrypt the early data.

**Figure 3:**
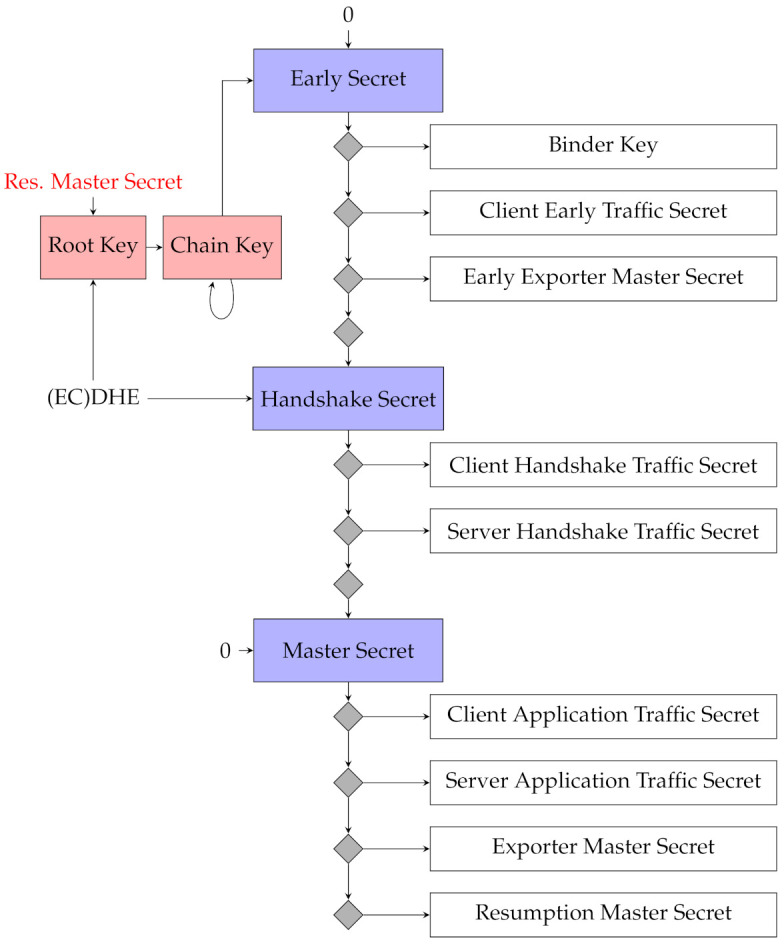
The rTLS key schedule. Red indicates added KDF instances. Blue indicates a default TLS HKDF instance. Grey diamonds indicate applications of the KDF function to produce a key.

When a DH handshake occurs during the resumption handshake (i.e., a key_share extension is included by both parties), the shared DH secret is used to derive all subsequent secrets for a TLS session as specified in the key schedule [1]. The TLS key schedule is included in Figure 3, with rTLS additions marked in red. This figure is an adaption of the one included in RFC 8446 [1]; for details on the key schedule itself, we refer the reader to the RFC. If a resumption secret already exists (e.g., because this is not the first session resumption), then the derivation will depend on both the existing resumption secret and the DH shared secret. This produces a new master secret, which (as per the key schedule) eventually generates a new resumption master secret, as can be seen by following the arrows in Figure 3. Whenever a DH handshake occurs, the ratchet index *must* be reset to 0, as the inner ratchets will be completely reset. Additionally, this makes it harder for a Man In The Middle (MITM) adversary to replace the client’s shared_key field with its own parameters, as it will also need to know the existing resumption secret, implying it would need to have access to either client or server already. Note that, when no DH handshake is performed, the ratchet Root Key is not updated at all. Instead, the Chain key feeds into itself (a ratchet step) and into the Early Secret.

Since we expect that, for virtually every scenario, one will want to reset the ratchets well before 255 communication attempts have been made, we only reserve 1 byte for the ratchet index. Additionally, when the ratchet index hits 255, we require both parties to delete their PSK and negotiate a new PSK with a standard handshake.

### 3.3. Double Ratchet Setup and Operation

Next, we summarize the extra steps needed for both the initial and resumption handshakes in a step-by-step fashion.

#### 3.3.1. Initial Handshake

The initial handshake is largely unmodified, but some special steps have to be taken by both the client and the server.

1. **ID generation**: The server generates a globally unique connection ID. This ID is transmitted to the client in the NewSessionTicket, together with a DH public key that the Client can use to initialize future resumption handshakes.
2. **Symmetric ratchet initialization**: The client and server initialize the ratchet index variable to 0. The symmetric ratchet root key is the resumption master secret.
3. **Persistent state storage**: Both client and server store their state variables for anticipated session resumptions.

#### 3.3.2. Resumption

Below we describe the extra steps needed for a typical session resumption. A DH exchange may take place, but we do not consider that as an extra step—the TLS standard already accommodates for this.

                                   **Client**

1. **Ratchet step**: The client ratchets its symmetric ratchet before the resumption master secret is used to derive any other secret. Thus, the early-data secret is derived from the ratcheted master secret.
2. **PSK exchange**: During the handshake, the client sends its ratchet index and connection ID to the server, as part of the pre_shared_key. If a DH exchange happens, the ClientHello includes a key_share structure, as well.

                                   **Server**

1. **Access state:** The server receives a 0-RTT resumption, and after having verified the pre_shared_key’s HMAC field, finds the relevant state variables using the received connection ID as a key (e.g., in a hash map).
2. **Replay condition** The server ensures that $i_{s}<i_{c}$, where $i_{s}$ and $i_{c}$ are, respectively, the server received client ratchet indices for this connection.
3. **Ratchet step**: The server spins the symmetric ratchet $i_{c}-i_{s}$ times, where $i_{c}$ is the received ratchet index in pre_shared_key, and $i_{s}$ its own ratchet index. The early data encryption key is derived from the new state of the sym. ratchet.

                                    **Both**

1. **Reset ratchet index**: If a DH exchange was performed during the resumption handshake, then the client and server reset their ratchet index to 0.
2. **Persistent state storage**: Both the client and server store their state variables for future session resumptions.

### 3.4. Ratchet State Variables

This extension expects both the client and server to maintain some state for each connection. This state consists of the following data:

1. **Mapping**: a connection ID → ratchet mapping, to identify which ratchet belongs to which connection. We set the connection ID to be 4 bytes in size as an initial estimate. It can be increased if necessary.
2. **Ratchet Index**: To indicate the number of ratchet steps that occurred since the last DH exchange (1 byte).
3. **Private DH key**: Current private DH key, used to compute a DH secret from which a common root key can be derived (32 bytes).
4. **Remote public DH key**: Last received remote public DH key for deriving aforementioned secret (32 bytes). Additionally, the Client and Server are expected to keep track of the Resumption Master Secret. We do not list it with the above state variables as this is something that already comes with standard TLS, thus not being unique to rTLS.

## 4. Security Evaluation

In this section, we discuss and analyze the security properties of the rTLS protocol extension. We formally define the intruder model, and then present a formal model of the rTLS protocol extension itself. Various security properties are automatically verified by the OFMC software, thereby giving us high certainty that they hold for the protocol, as well.

### 4.1. Formal Verification

Now, we present a formalization and verification in OFMC [7], a tool for formal verification of security protocols. It uses a symbolic Dolev-Yao-style model of cryptography, i.e., messages are represented in a term algebra where the algebraic properties of operators are represented (e.g., the properties of exponentiation needed for Diffie-Hellman). It formalizes a state-transition system through multi-set rewriting rules, and the main technique is a constraint-based representation of the intruder, dubbed the lazy intruder, which allow for verification without bounding the number of steps that the intruder can perform. However, the steps that the honest participants can perform needs to be bounded (or the tool will not terminate, in general). This choice of formal analysis software is motivated by the fact that most tools, such as ProVerif and Tamarin, run into problems with the ratchets since in an unbounded number of sessions, this creates structures for which the usual abstractions and bounding lemmata fail, but they do work in OFMC due to the bounds, allowing us to express the ratchets without problems. There are several input languages for OFMC, the native one being the AVISPA Intermediate Format IF [15] based on set-rewriting (similar to the input language of Tamarin). This can be considered kind-of a “protocol assembly language”, i.e., it is hard to write by hand. The high-level languages available are Alice-and-Bob-style language AnB, but this language is too limited to express ratchets. There is also the AVISPA [16] High-Level Protocol Specification Language HLPSL [17] and its successor ASLan from the AVANTSSAR project [18]. Both languages would be suitable for our purposes, but the updating of local states that we have to perform make them not much more easy for the specification than IF, so we directly relied on IF for an initial formal verification [19]. We have, however, inspired by this work, developed a more high-level notation for protocols of this style and are currently working on a general compiler from this notation to IF to benefit in similar projects from it. We will use this high-level notation in the following presentation to explain our formal model.

#### 4.1.1. Intruder Model

We define two roles, Client and Server. Each role can, in principle, be instantiated arbitrarily often by any number of clients and servers. We need to limit this for OFMC to two sessions, albeit symbolic ones, meaning that the name of the client and the server is a variable where the intruder can determine who is playing. Thus we include at all kinds of two-session scenarios, e.g., an honest Alice as client with the intruder as server in parallel with a session between honest Alice and Bob as client and server. Note that the intruder can play any of the roles under his real name, where he has access to appropriate initial key material shared with a client or server; the payload messages exchanged in such a session are of course not secret. To allow the intruder to participate as a “normal” agent is essential to capture attacks where an intruder is, for instance, a dishonest server contacted by an honest Alice, and uses part of the messages from this session to attack another session, as in the famous Needham-Schroeder PublicKey Protocol (NSPK) attack [20].

In the style of the Dolev-Yao intruder model, the intruder also controls the network, i.e., every message an honest agent sends goes to the intruder, and every message an honest agent receives comes from the intruder. The intruder can perform normal cryptographic operations with keys he knows, just as any other agent.

The starting point is that a Client and Server have successfully established a secure TLS 1.3 session in the past and, thus, share a resumption master secret; moreover, the Client has obtained a session ticket containing a DH public key, as well as connection ID from the Server.

#### 4.1.2. Resumption Handshake Model

Next, we present a detailed model of the resumption handshake protocol. This is effectively the standard TLS 1.3 0-RTT resumption protocol, with early data protected through a rotating (ratcheted) key.

First, every session of an agent is characterized by a number of state variables that are updated during the course of the session. These are shown in Table 2. Both share the same resumption master secret (RES_MASTER_SECRET) and connection ID (CONN_ID). In OFMC, we model this by a secret function resMasterSecret(C, S, CONN_ID) that, for a given client name, servername, and connection ID, returns a unique strong key; the intruder is given all keys where he is *C* or *S*. The root key *RK* is derived from RES_MASTER_SECRET. Note that CONN_ID is simply a unique identifier.

Both the Client and Server store the latest DH public key received from the other side as ServerDHsPub and ClientDHsPub, respectively. They also store their own latest DH private key currPrivate. Because the Client has received a DH public key from the server during the first session in a NewSessionTicket, we assume that ServerDHsPub and the Server’s currPrivate are initially populated. In OFMC, we model the initial private key of the server again with a private function secret_exponent(S, CONN_ID) for the server (known to the intruder whenever S=i).

Finally, the Client needs to store its sending chain and the server needs to store its receiving chain. These consist of a chain key and a chain index, which are defined as ClientCKs and ClientNs for the Client, and ServerCKs and ServerNs for the server. However, these do not need to be initialized at the start. The Client will compute its private key before transmitting the first resumption message.

Similar to the session bounding in OFMC, we also need to bound how many ratchet turns each agent can make in each session. Again we have to limit ourselves to a quite low bound of 2 repetitions, but this should cover all likely scenarios. As a modeling trick, we just initialize both counters with 2, and, in each resumption, we decrease until it is 0.

#### 4.1.3. Step 1: ClientHello

Now, we use the state variables to construct a detailed description of an execution of the 0-RTT protocol. Note that all steps come in two variants: with a new DH key exchange and without. In the OFMC implementation, the client can choose which variant. We describe only the variant in detail that does the DH key exchange, and we only mention the difference when no DH key exchange is done.

If a DH key exchange is to be included in the handshake, then, the first action is that the Client generates a new private key, as well as a shared DH key, together with the Server’s DH public key. When the Client has computed this DH secret, it passes the key into its inner ratchet, by applying the KDF function on the DH secret combined with a root key RK, and, finally, obtains the ClientCKs:

new currPrivate

RK     := bkdf(RK,exp(ServerDHsPub,currPrivate))

ClientCKs := kdf(RK)

where we use kdf and bkdf to model the corresponding key derivation functions.

Now, we can describe the initial message sent from a Client. First, it spins its ratchet and increases ClientNs by one (i.e., actually in the OFMC model, decreases, if not yet zero). The key generated through this is used as input material for the Early Secret in the TLS key schedule. We focus only on the relevant parts of a resumption ClientHello message, specifically, the early data itself (MOUT, TLS session ID, client randomness and the relevant resumption parameters. The keys $K_{1}$, the client_early_traffic_secret and $K_{2}$, the binder_key are derived from the Early traffic secret as can be seen in Figure 3. At this point, it is important to note that since the Client can choose to include an optional key_share extension (DH handshake) in the ClientHello, the inclusion of ClientDHsPub in the resumption handshake is also optional. The early data is encrypted with client_early_traffic_secret. Additionally, the plaintext data is integrity protected through a MAC with key binder_key. Both keys are derived from the master key conform the TLS standard:

let MSG1=step0(ClientNs,exp(g,currPrivate),CHR)

let K1=hkdf(ClientCKs,MSG1)

let K2=hkdf(ClientCKs,pair(exp(ServerDHsPub,currPrivate),pair(C,S)))

send(step1(scrypt(K2,MOUT),hmac(K1,MSG1),MSG1))

Here, $step_{0}$ and $step_{1}$ are message formats that represent how the cleartext data is serialized (i.e., every agent, including the intruder, can compose and decompose such messages without any keys). hkdf is another key-derivation function, pair stands for pure string concatenation, and scrypt(k,m) stands for symmetric encryption of message *m* with key *k*, and hmac(k,m) stands for a hash-mac with key *k* of message *m*.

When the Server receives a ClientHello with early data indication, it first has to spin its inner ratchet to derive an early_secret identical to that of the Client. Included in this step is the incrementing of ServerNr. The Server can then derive the keys necessary to authenticate and decrypt the received early data. After this point, the Server proceeds differently based on whether the key_share extension was included by the Client. If the extension was not included, the Server continues using the current chain for future resumptions and can simply continue the current handshake as usual. If the extension was included, the Server will have to spin its DH ratchet, as well, which, in turn, leads to an update of the Server’s receiving chain root key. Note that this new DH secret is not just for future sessions and is already used in the remainder of this handshake as it normally would be in a TLS session, as we explain in the next paragraph.

In the high-level notation, we have: 

receive (step1(SM2,HM1,M1))

try   step0(SN,ClientDHsPub,CHR)==M1

let  DH   = exp(ClientDHsPub,currPrivate)

RK      := bkdf(RK,DH)

ServerCKr  := kdf(RK)

let  K1=hkdf(ServerCKr,M1)

try  HM1==hmac(K1,M1)

let  K2=hkdf(ServerCKr,pair(DH,pair(C,S)))

try  MIN==dscrypt(K2,SM2) style=beautIF

Note that the try  is used to describe operations that might fail, such as trying to decrypt, parse, or check for equality. When it fails, the agent simply does aborts the transaction and rolls back to the state before the transaction. In particular, the first try  in the above code snippet parses the received message M1 as the step0 format, extracting the three components of the message. The next try  is checking that the received hmac HM1 is the same as constructing hmac(K1,M1), and the last try  is trying to decrypt the message SM2. Note that we assume here symmetric encryption with MACs that tells us if decryption succeeded. Observe the contrast to the let    x=t command, which simply means replacing all further occurrences of x with t, and the x:=t command, which means that the state variable x is set to t.

#### 4.1.4. Step 2: ServerHello

Next, the Server will reply with a ServerHello message. If the Client included a key_share extension, then the server will reply with its newly generated DH public key from the previous step. Before the response can be sent, the Server has to compute all the remaining keys from the TLS key schedule. This starts with computing the handshake_secret. The KDF function for this secret takes two inputs, one being the hash product of the previous phase in the key schedule, and another being fresh Input Key Material (IKM). If a DH handshake occurred, then the resulting DH secret should be used as IKM here. If no DH handshake occurred, the IKM is simply set to 0. The ServerHello response itself includes a number of fields which are not relevant for our verification, so we leave them out. We do include EncryptedExtensions (EE) as a representative message payload and the contents of the Finished message type, which has a field verify_data, containing an HMAC of the handshake context. This HMAC protects the integrity of ServerDHsPub and Server_rand, as well; therefore, we add these to the encrypted payload, while leaving other parts out to keep the model concise. We can do this, as the HMAC key is directly derived from the server_handshake_traffic_secret. We include Server_rand, as this is 32 bytes or randomness that is used for various cryptographic purposes and acts as a nonce. Finally, the Server has the opportunity to already send application data (App_Data) with its response.

Different parts of the transmission are encrypted with different keys derived from the master secret conform the TLS standard. The remainder of the handshake, i.e., most of the ServerHello message is encrypted with the server_handshake_traffic_secret. If the Server chooses to include a response payload, then this optional response can already be encrypted with the server_application_traffic_secret.

new   currPrivate

new  SHR

let   DH     = exp(ClientDHsPub,DHs)

RK         := bkdf(RK,DH)

ServerCKr    := kdf(RK)

let   K2=serverK(hkdf(DH,pair(ServerCKr,pair(CHR,pair(C,S)))))

let   MSG2=scrypt(K2,pair(exp(g,DHs),SHR))

let   K1=serverK(hkdf(DH,pair(ServerCKr,pair(SHR,pair(CHR,pair(C,S))))))

let   MSG1=scrypt(K1,MOUT)

send (step2(MSG1,MSG2,SHR,exp(g,DHs)))

When the Client receives the Server’s ServerHello, it first has to continue with its own execution of the TLS key schedule. If the Client initiated with a new DH public key and, thus, a key_share extension, the server replied with a fresh DH public key in its own key_share. This is then used by the Client as input for the handshake secret identically to how the server processed the DH secret. With this, the Client can continue the TLS key schedule until all keys are derived. Note that, for both the Server and Client, the newly computed Resumption Master Secret is assigned to the inner chain’s root key, but not necessarily included in the current chain; if no DH handshake was included, the inner chain is not reset. This does not matter, as no new entropy was introduced during the handshake either way. As is evident from the description of the operations of the rTLS resumption process given in this section, the optional DH exchanges feed into the TLS keyschedule and provide new entropy that gets propagated through to the inner chains and as a result future executions of the key schedule.

receive (step2(M1,M2,SHR,ExpgDHs))

let  DH=exp(ExpgDHs,currPrivate)

ServerDHsPub    :=ExpgDHs

RK     :=bkdf(RK,DH)

ClientCKs:=kdf(RK)

ClientNs  :=s(ClientNs)

let K2=serverK(hkdf(DH,pair(ClientCKs,pair(CHR,pair(C,S)))))

try  pair(ExpgDHs,SHR)==dscrypt(K2,M2)

let  K1=serverK(hkdf(DH,pair(ClientCKs,pair(SHR,pair(CHR,pair(C,S))))))

try MIN==dscrypt(K1,M1)

#### 4.1.5. Step 3: Finished

The Client finishes the 0-RTT handshake with an EndOfEarlyData message and a Finished message. The EndOfEarlyData message is simply an indicator that the Client has no more early data to transmit and that all future data will be encrypted with the client_application_traffic_secret.

let  TMP=pair(pair(C,S),pair(ExpgDHs,pair(CHR,SHR)))

let  K3=clientK(hkdf(DH,pair(ClientCKs,TMP)))

send (scrypt(K3,MOUT))

#### 4.1.6. Verification

We verify a number of security goals, the first of which is secrecy. We want the early data, i.e., MOUT/MIN payloads, to be secret between Client and Server. The second security goal we verify is injective agreement [21]. This means that, when an honest party *B* receives a payload message apparently from *A*, then, either *A* is the intruder under his real name (no authentication guarantees) or *A* indeed sent that payload message for *B* (and they agree on all roles). Moreover, this is injective in the sense that *B* does not accept the same payload more often than it was sent by *A*, so there is no replay.

Using OFMC, we verify the described properties to hold for the rTLS resumption protocol. Due to an exponential increase of the search spaces with the number of sessions and resumptions, we bounded the number of sessions to 2, and the number of resumptions in each session also to 2. Note, however, that we have here symbolic sessions, i.e., they can be arbitrarily instantiated, including with the intruder as a client or server. Moreover, in each session and resumption, the client can decide to either perform a new DH key or not. We also extensively tested the specification, namely that all expected steps could be taken, in particular that honest agents can communicate, and the intruder can play each of its roles under his real name as a normal participant.

OFMC reported that no attacks were found in any runs which gives a high assurance that the rTLS session resumption protocol provides secrecy and injective agreement: While this is only proved for 2 sessions and with 2 resumptions each, it seems unlikely that further sessions and resumptions would allow for additional attacks because of the symmetry of all further repetitions.

Finally, we want to look at the so-called selfie attack [22]: this is an attack that works on some pre-shared-key deployments of TLS 1.3, where a client *C* and server *S* use the same pre-shared key psk(C,S)=psk(S,C) in both directions of communication, allowing for reflection attacks. Similarly, if we allow in our rTLS model:

RES_MASTER_SECRET(C,S,CONN_ID)=RES_MASTER_SECRET(S,C,CONN_ID),

then we still do not get a selfie-attack because the setup of the Diffie-Hellman ratchet is different for client and server role. This is, however, looking only at the initial state of the resumption handshake rTLS, not at the preceding steps of the original TLS. This means that, if the setup of TLS is such that it does not allow for a selfie attack, then, by construction, rTLS cannot induce a selfie attack either.

## 5. Performance Evaluation

In this section, we present numeric estimates of the performance of rTLS, with as performance indicators traffic overhead and storage overhead. The numerical data is based on the estimated data structure size of the state variables and TLS message structures as they are defined in the TLS standard.

### 5.1. Traffic Overhead Estimation

#### 5.1.1. Initial Handshake

The rTLS initial handshake does not differ in traffic overhead from a normal TLS handshake, since the only change defines an extra value for an enumerated field, which is (psk_key_exchange_modes). After finishing the handshake, the server transmits a NewSessionTicket message to the client. While technically not part of the initial handshake, we consider it as such in this context; without it, resumption would not be possible. The structure and size of a minimal NewSessionTicket message are displayed in Table 3. Here, |X| indicates the size in bytes of element *X*. The client does not need to send any reply to this message. The ticket field itself has to contain the connection identifier, as well as a public DH key that the client can use for the first resumption; so, we set the size of the ticket field to 4+32 bytes, and we include a 32 byte nonce, as well. We do not need to explicitly include a ratchet index here, as it can be initialized to 0 by both parties. Therefore, compared to no session resumption at all, minimal overhead is 14+36+32=72 bytes. Compared to a session ticket in standard TLS 1.3, which, in OpenSSL, is typically around 528 bytes, this is a significant improvement of 86 percent.

#### 5.1.2. Resumption Handshake

It is important to reduce traffic overhead for the resumption handshake as much as possible, since this will typically be performed much more often than an initial handshake. The minimal cost for any resumption handshake consists of boilerplate parts of the handshake that cannot be eliminated without rigorous change to the TLS protocol. In the following, we write client and server as *c* and *s*, respectively. We map symbols to every message element in the resumption handshake in Table 4, where *x* can be either *c* or *s* to indicate the message sender.

We define the minimal traffic overhead cost *C* of any 0-RTT resumption handshake as:

(1) $$C=3|R|+|H_{c}\left|+\right|H_{s}|+|ed_{c}|+|pex|+|ee|+2|f|+|eed|.$$

This cost is not a fixed number of bytes but, rather, is not negotiable; any PSK extension will have to include these elements, and their size is independent of the actual PSK mode. The total cost of a minimal resumption handshake is then $C+|psk_{c}|+|psk_{s}|$. Note that $ks_{c}$ and $ks_{s}$ are not required for a minimal handshake. Conforming to the standard, $psk_{s}$ is defined as a 2-byte value representing an identity index in $psk_{c}$ and is wrapped in a 4-byte TLS extension structure. However, $ks_{c}$ is more complex, and we write the full layout in Table 5. Note that the term “identifier” here refers in the standard to the ticket field itself, but we use it to transmit a concatenation of the connection identifier and ratchet index. Because we only send one identity and binder, The size of $psk_{c}$ becomes $|psk_{c}|=15+\alpha +\beta$, where α denotes the size of the identity field, and β the size of the binder HMAC. The identifier field PSKID can be written as PSKID=ID||i, where ID is the identifier received in the session ticket during the initial handshake, and *i* is the symmetric KDF chain index. Now, |PSKID|=|ID|+1=5. The exact value of β depends on the chosen HMAC function, which is usually either Secure Hash Algorithm (SHA)-256 (32 bytes) or SHA-384 (48 bytes). The complete traffic cost $c_{1}$ for session resumption can, thus, be written as $c=|psk_{s}|+|psk_{c}|+C=26+\beta +C$, and it is 58+C if SHA-256 is chosen.

When a DH exchange is included, we will have to add the size of the $ks_{c}$ and $ks_{s}$ elements. The size of $ks_{c}$ is of variable length depending on the number of supported DH groups the client advertises. Each key share entry takes up 4+l bytes, where *l* is the size of the supported group. The smallest supported group is X25519 with a 32-byte field, while the largest is P-521 with 132 bytes. $ks_{c}$ also reserves 2 bytes to denote the number of listed groups. If we only transmit one group, the size is, therefore, $ks_{c}=6+l$. The server replies with a single key share entry; thus, $ks_{s}=4+l$. As with any TLS extension, both $ks_{s}$ and $ks_{c}$ are wrapped in an extension structure with a 4-byte type field. The total cost of a resumption with DH exchange is, thus, $c_{2}=c_{1}+|ks_{c}|+|ks_{s}|=c_{1}+18+2l$.

**Figure 4:**
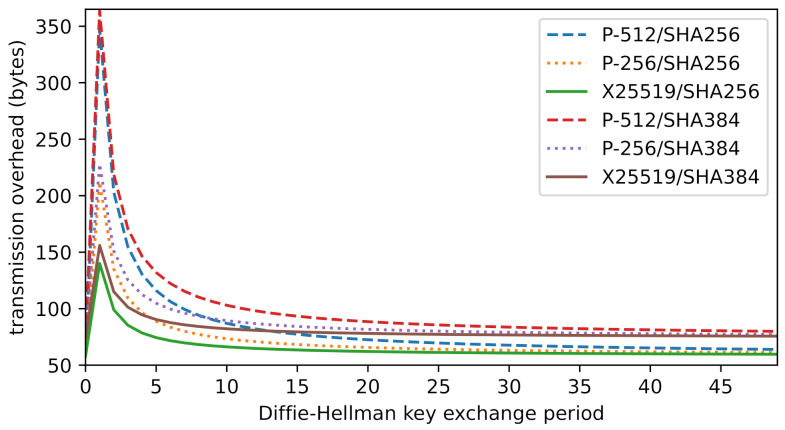
Average transmission overhead versus DH key exchange period.

If we take into account a key_share every *n* messages, we arrive at the final equation for the total average cost $c_{t}$:

(2) $$\begin{array}{c} c_{t}=\left\{\begin{array}{cc} 26+\beta +C & \mathrm{for}\;n=0\\ 26+\beta +\frac{18+2l}{n}+C & \mathrm{for}\;n>0 \end{array}\right\}, \end{array}$$

where β is the hash digest size, *l* the elliptic curve coordinate length, *n* the DH handshake rate, and *C* the minimal cost. Figure 4 shows the average overhead (i.e., without *C*) versus the key exchange period, for various common cipher suites.

Giving an exact value for *C* is somewhat difficult: multiple fields in $H_{c}$, $H_{s}$, and ee can vary a lot in length, depending on the supported cipher suites and provided extensions among other things. Instead, we count the minimum size for these fields as they are defined in the standard, thereby giving a lower bound for *C*. Note that, in practice, a handshake with so few extensions is not useful for overhead minimization, as more round-trips will be needed to establish necessary parameters, such as the cipher suite. Moreover, it leaves out extensions meant to increase overall security. Minimal sizes, including all headers, for $H_{c}$ and $H_{s}$ are 50 and 48 bytes, respectively. $ed_{c}$ and eed both require 2 and 4 bytes. pex is, at least, 3+m bytes in size, where *m* is the number of supported modes (at least 1). ee is, at least, 6 bytes in size but may vary a lot, depending on the supported extensions. The length of *f* is determined by the chosen hash function. The record layer headers are 5 bytes in size. With one PSK key exchange mode and the SHA-256 hash function, the total cost of *C* is then, at least, 193 bytes. Therefore, the lower bound on transmission overhead of a resumption handshake with our extension is 251 bytes without, or 333 bytes with a key exchange. If we include several extensions for a more realistic minimal handshake, we can expect the cost to be between 400 and 600 bytes.

### 5.2. Storage Overhead Estimation

Both the client and server need to store some state variables in between sessions. This differs from the standard session resumption protocol where only the client stores the PSK. The client needs to securely store the secret KDF key (depends on digest size), as well as its connection ID (4 bytes) and the ratchet index (1 byte). Additionally, the client needs to keep track of its current DH private key and the last received DH public key from the server, the size of these depends on the chosen group. Thus, the client needs to store 101 bytes if SHA-256 and X25519 are used.

The server needs to store the same amount of state, but for every client that it shares a ratchet for resumption with. This can be done through, e.g., a hash map using the connection ID as a key, and a structure containing the other state variables as value. If state is being kept for the maximum amount of clients of $2^{32}$ (with a 4-byte connection ID), this amounts to roughly 433 GB worth of data. When there is a large set of clients connecting to the server and, thus, a large amount of state variables, one should be mindful of access times and pick data structures that minimize access time, such as hash maps, to provide some protection against denial of service attacks.

### 5.3. Overhead Comparison with TLS 1.3

Based on measurements performed on OpenSSL [6], a standard PSK in TLS 1.3 adds 571 and 603 bytes of overhead, respectively, when SHA-256 SHA-384 is used. In Table 6, we compare the overhead of rTLS for various key exchange periods *n* to that of a standard TLS 1.3 PSK. We use a higher value of 408 for *C*, obtained from handshake measurements in OpenSSL, which includes a minimal number of extensions by default, and acts as an indicative value that represents a lightweight use case. In this table, the values are computed using the smallest allowed hash function (SHA-256) and curve (X25519). As can be seen, a rTLS PSK requires only roughly 11% of the traffic overhead compared to a standard TLS PSK and can be expected to reduce the total amount of transmitted data roughly by half.

## 6. Related Work

There exist ample communication security protocols aimed at embedded devices [23]. We look at the TLS protocol and its variants, specifically those that are relevant to the usage of this protocol in embedded environments. We also briefly look at QUIC.

Initially developed for Web security, TLS is now gaining traction in the IoT world, partly due to widely available libraries and broad support in software relevant to IoT. For example, many Message Queuing Telemetry Transport (MQTT) brokers support TLS as a security layer.

While this is fine for most devices (mostly upwards from class 1 in the IETF classification [24]), it becomes problematic when working with class 0 or low-end class 1 devices, as they do not possess the capability to maintain TLS connections or can simply not afford it due to resource constraints (e.g., due to a power budget). To address this, several optimizations have been proposed over the years. One of the first was Sizzle [25], which is an implementation of the Secure Socket Layer (SSL) protocol, capable of running on extremely constrained devices with only tens of kilobytes of memory. While the authors showed that heavyweight cryptographic operations required for the protocol to function were certainly possible on heavily constrained devices, they did not attempt to reduce the amount of transmitted data.

Datagram Transport Layer Security (DTLS) [26] modifies the TLS protocol to work over User Datagram Protocol (UDP), while retaining most of the security guarantees provided by TLS. This reduces the data overhead and latency somewhat. Recently, the DTLS 1.3 draft [27] was approved by the IETF. This revision brings 0-RTT and other TLS 1.3 improvements to DTLS. There exist multiple open-source implementations [28], and several works exist detailing extremely lightweight implementations [29,30]. In these works, lightweight mostly pertains to computational and memory cost, while transmission overhead is either not addressed or addressed to a much lesser degree. Other approaches have been taken, as well, such as Reference [31], compressing DTLS messages to fit into 6LowPAN frames. Recently, a performance comparison of TLS 1.3 and DTLS 1.3 on lightweight IoT devices was published [32], showing that, while both TLS and DTLS 1.3 add suffer from larger overhead in terms of memory usage and transmission overhead, these are within bounds for these protocols to be used on devices that can already run the 1.2 version. Additionally, the authors state that there is room for optimizations in software to further reduce the overhead. In Reference [33], a DTLS fast session resumption mechanism is proposed, making use of free UDP ports on the server-side. However, the proposed protocol does not address forward security and provides no analysis of its security claims.

While DTLS has less bandwidth overhead than TLS, it is still not ideal for lightweight scenarios with message proxies (e.g., brokers, such as in MQTT). To address this, the recently standardized application-layer Object Security for Constrained RESTful Environments (OSCORE) protocol aims to enable selective encryption of parts of the Constrained Application Protocol (CoAP) protocol. Gunnarsson et al. [34] show that this provides a slight performance improvement over the default DTLS security option. Due to OSCORE’s selective encryption approach, it can provide end-to-end encryption in situations where messages are relayed through proxies, whereas TLS-based protocols have to setup separate secure channels between each proxy. However, when no proxies are needed, TLS-based protocols might offer better performance especially when 0-RTT is taken into account.

Several extensions for TLS have been proposed that also bring the potential to lower message overhead. The TLS Cached Info specification [35] allows clients to store server certificates and certificate requests, making it possible to leave these out in future handshakes. The TLS Raw Public Key extension [36] allows clients and servers to authenticate each other through public keys, instead of X.509 certificates. This can significantly reduce the handshake size. This method does require an out-of-band means of verifying public keys, which might very well be possible in a controlled environment, such as a factory. Another promising adaptation of TLS that might lower the size overhead of TLS significantly is the Compact Transport Layer Security (CTLS) IETF draft [37]. In this draft, the authors propose optimizing the TLS protocol for size by eliminating redundancy where possible and making aggressive use of space-optimization techniques, such as variable-length integers. The result is isomorphic to TLS, but not interoperable.

Additionally, in our previous work [5], we introduced rTLS, a TLS 1.3 protocol extension that focuses specifically on the 0-RTT session resumption protocol, with the goal of making it more usable for the IoT. In our original work, we presented the protocol and included numerical estimates on its performance but did not include a thorough analysis of its security properties. In this work, we extended upon that and present a formal security analysis, as well as some fixes to the protocol that were overlooked in the original work. The rTLS extension is compatible with the aforementioned cTLS draft, as well as other TLS extensions. For DTLS, it is very likely that some adjustments are necessary as the DTLS resumption protocol is slightly different.

DTLS is also proposed as the default mechanism to secure connections in the QUIC protocol, a network protocol building on UDP that provides advanced features, such as multiplexing and authenticated encryption of its data by default.

Session resumption in TLS 1.3 has been subject to debate, as it is vulnerable to replay attacks and provides no forward secrecy [1]. While, for a Web environment, there exists some justification for these design choices, for an IoT environment where short conversations with short messages are the norm, this is less than ideal, as it effectively removes the possibility to optimize overhead through use of the session resumption protocol. None of the extensions discussed in this section address session resumption, which means that this is an open issue we think has significant potential for minimizing protocol overhead, when designed carefully.

At the time of writing, National Institute of Standards and Technology (NIST) is hosting an ongoing competition for lightweight cryptographic primitives [38]. Many of the candidates specifically target very short messages. Once the candidates have received sufficient cryptanalytic attention, these can become valuable tools in future lightweight communication protocols, as well as potentially helping protocols, such as TLS adapt to constrained devices.

In Reference [39], Hall-Andersen et al. acknowledge the complexity of TLS and propose nQUIC as a lightweight, less complex alternative to QUIC’s default TLS configuration. Their experiments show a significant reduction in bandwidth compared to TLS.

## 7. Conclusions

In this work, we extended upon an IoT-friendly and standard-compliant adaption of the TLS 1.3 0-RTT session resumption protocol. We first argued that, in order to be applicable to IoT, replay resistance is a necessary property, as lightweight sensor devices are much more likely to transmit data that will change server state.

Building from the observation that, in IoT scenarios, the group of possible clients for a server changes relatively slowly and is typically much smaller than possible clients for a Web server, we argued that it is reasonable to require a server to keep some state variables for each of its clients. We then took inspiration from the Double Ratchet algorithm to design a 0-RTT resumption protocol that fits neatly into the existing message structure, and makes use of existing functionality where possible. In our extension, the PSK utilizes a ratchet construction, which provides replay protection, as well as forward secrecy and break-in resilience to early data transmitted in a 0-RTT handshake. The introduction of these properties in the 0-RTT sub-protocol is a step toward making TLS suitable for IoT scenarios.

We estimated a lower bound of 193 bytes on traffic overhead for any 0-RTT resumption protocol in TLS 1.3 and then showed that a resumption handshake with our protocol would result in around 466–516 transmitted bytes, depending on the chosen DH key exchange period. Compared to the standard session resumption transmission size of roughly 979 bytes, this is a significant improvement.

Extending our previous work, the protocol received minor updates relating to what state should be kept. Additionally, we improved the presentation of the protocol, including a more detailed description of how the TLS key schedule is affected. These minor changes are also propagated into the performance evaluation estimates, affecting mainly the storage overhead estimates. We also added a new section detailing a formal security analysis of the protocol in the Dolev-Yao model. The results of this analysis give high assurance that the protocol provides secrecy, as well as security against replay attacks.

In future work, we aim to further reduce the transmission overhead by exploring different opportunities, such as replacing the original message structure for resumption altogether, thereby reducing the fixed cost. Moreover, we are currently testing a proof-of-concept implementation to support the overhead estimations with empirical results.

## Figures and Tables

**Table 1:** Layout of the NewSessionTicket structure.

Type	Field Name	Description
uint_32	ticket_lifetime	ticket lifetime in seconds
uint_32	ticket_age_add	used to obscure ticket age
opaque	ticket_nonce	(max. 255 bytes) nonce
opaque	ticket	(max. $2^{32}$ bytes) ticket itself
Extension	extensions	(max. $2^{32}$ bytes ) extensions

**Table 2:** The initial state for both client and server.

Client State		Server State	
State Variable	Initial State	State Variable	Initial State
RES_MASTER_SECRET	from TLS	RES_MASTER_SECRET	from TLS
*RK*	…	*RK*	…
CONN_ID	from TLS	CONN_ID	from TLS
*ServerDHsPub*	$g^{X}$	*ClientDHsPub*	-
*currPrivate*	-	*currPrivate*	*X*
*ClientCKs*	-	*ServerCKr*	-
*ClientNs*	0	*ServerNr*	0
*CHR*	-	*CHR*	-
		*SHR*	-
*Step*	0	*Step*	0

**Table 3:** The message structure and size of a minimal NewSessionTicket message. Here, |T| refers to the ticket length, and |N| to the size of the nonce.

	Size (Bytes)	Field Name
	4	ticket_lifetime
	4	ticket_age_add;
	|N|	ticket_nonce
	|T|	ticket
	2	extensions length
	4	Early data extension
**Total**	14+|T|+|N|	

**Table 4:** Symbol definitions for message elements, where x∈{c,s} refers to the message sender (client resp. server).

Symbol	Description
$H_{x}$	(Client or Server) Hello
$ed_{x}$	early_data
$D_{x}$	Application data
pex	psk_key_exchange_modes
$psk_{x}$	pre_shared_key
$ks_{x}$	key_share
ee	EncryptedExtensions
eed	EndOfEarlyData
*f*	Finished
*R*	Record Layer headers

**Table 5:** Layout of the pre_shared_key structure and its sub-structures, when sent by a client.

pre_shared_key		
Size	Field Name	Description
2	extension_type	Extension type
2	extension_data	Size of the extension
2	PSKIdentities_length	Nr. of PSK identities
	identities	PSKIdentity values
2	binders_length	Nr. of PSK binders
	binders	PSKBinder values
**PSKIdentity**		
2	identity length	Size of identity field
α	identity	value of this identity
4	obfuscated_ticket_age	ticket age (see Reference [1])
**PSKBinder**		
1	binder length	size of the binder value
β	binder	HMAC value (see Reference [1])

**Table 6:** A comparison between rTLS session resumption and OpenSSL standard session resumption.

Indicative Lightweight Use (C=408)		
Scenario	Avg. Overhead (b)	Avg. Total Size (b)
rTLS, n=0	58	466
rTLS, n=1	108	516
rTLS, n=10	63	471
Standard TLS 1.3	571	979
